# Computation of Fresnel Integrals

**DOI:** 10.6028/jres.102.025

**Published:** 1997

**Authors:** Klaus D. Mielenz

**Affiliations:** Alpine Lake Resort, Terra Alta, WV 26764

**Keywords:** computation, Fresnel integrals, numerical approximations, series expansions, spreadsheet computations

## Abstract

This paper describes a method for spreadsheet computations of Fresnel integrals to six significant figures, based on successive improvements of known rational approximations which are accurate to only three figures. Outside the range of validity of the improved approximations, known series expansions are used to obtain the Fresnel integrals to six figures.

## 1. Known Formulae

The Fresnel cosine and sine integrals,

$$\begin{array}{l} C\left(x\right)\int_{0}^{x}dt\cos\left(\frac{\pi }{2}t^{2}\right)\\ =\frac{1}{2}+f\left(x\right)\sin\left(\frac{\pi }{2}x^{2}\right)-g\left(x\right)\cos\left(\frac{\pi }{2}x^{2}\right), \end{array}$$ (1a)

$$\begin{array}{l} S\left(x\right)=\int_{0}^{x}dt\sin\left(\frac{\pi }{2}t^{2}\right)\\ =\frac{1}{2}-f\left(x\right)\cos\left(\frac{\pi }{2}x^{2}\right)-g\left(x\right)\sin\left(\frac{\pi }{2}x^{2}\right), \end{array}$$ (1b)

where *f*(*x*) and *g*(*x*) are auxiliary functions, are important in various fields of physics. This author required them to six significant figures for a project in optics which employed spreadsheet software to compute and plot three-dimensional diffraction patterns. It was found that software and algorithms for computing the Fresnel integrals in Fortran and C are readily available [1, 2]. However, this software was not directly applicable in spreadsheets and the algorithms were more complicated than necessary for this project. It was therefore decided to develop suitable approximations that can be programmed into spreadsheet programs to supply the required values of C(x) and *S*(*x*).

This development was based on the following formulae listed in the NBS Handbook of Functions [3]:

$$f\left(x\right)\approx f_{0}\left(x\right)=\frac{1+0.926x}{2+1.792x+3.104x^{2}}+\varepsilon \left(x\right),$$ (2a)

$$g\left(x\right)\approx g_{0}\left(x\right)=\frac{1}{2+4.142x+3.492x^{2}+6.67x^{3}}+\varepsilon \left(x\right),$$ (2b)

$$\left|\varepsilon \left(x\right)\right|\leq 2\;10^{-3},\;0<x<\infty ;$$ (2c)

$$\begin{array}{l} C\left(x\right)=x-\frac{\pi^{2}x^{5}}{40}+\frac{\pi^{4}x^{9}}{3456}+\varepsilon \left(x\right),\\ \left|\varepsilon \left(x\right)\right|\leq 3\;10^{-6},\;\left|x\right|<0.63, \end{array}$$ (3a)

$$\begin{array}{l} S\left(x\right)=\frac{\pi }{6}-\frac{\pi^{3}x^{7}}{336}+\frac{\pi^{5}x^{11}}{42240}+\varepsilon \left(x\right),\\ \left|\varepsilon \left(x\right)\right|\leq 3\;10^{-6},\;\left|x\right|<0.73; \end{array}$$ (3b)

$$\begin{array}{l} f\left(x\right)=\frac{1}{\pi x}-\frac{3}{\pi^{3}x^{5}}+\frac{105}{\pi^{5}x^{9}}+\varepsilon \left(x\right),\\ \left|\varepsilon \left(x\right)\right|\leq 3\;10^{-6},\;x>2.9, \end{array}$$ (4a)

$$\begin{array}{l} g\left(x\right)=\frac{1}{\pi^{2}x^{3}}-\frac{15}{\pi^{4}x^{7}}+\frac{945}{\pi^{6}x^{11}}+\varepsilon \left(x\right),\\ \left|\varepsilon \left(x\right)\right|\leq 3\;10^{-6},\;x>2.8. \end{array}$$ (4b)

As the rational approximations of Eqs. (2a) and (2b) yield *C*(*x*) and *S*(*x*) only to ±2.5 × 10^−3^, it was necessry to improve them over a sufficiently wide range of intermediate arguments so that they could be supplemented with the series expansions of Eqs. (3a) to (4b) to compute *C*(x) and *S*(*x*) to ±3 3 10^−6^ for arbitrary values of *x*.

## 2. Improvement of Rational Approximations

The improvement of the rational approximations of Eqs. (2a) and (2b) was based on a comparison of the numerical values of *f*_0_(*x*) and *g*_0_(*x*) to the exact values tabulated in Ref. [1]. It was found that the residuals *ε*(*x*) were skewed oscillatory functions that could be approximated by expressions of the form *ε*(*x*) ≈ Δ(*x*) = *a*(*x*) sin[*α*(*x*)] + *b*(*x*), where *a*(*x*) and *b*(*x*) are linear functions of *x* and *α*(*x*) is chosen so that the oscillations of *ε*(*x*) are imitated. By a judicious choice of parameters it was arranged that the second residuals, *ε*(*x*) − Δ(*x*) also exhibited a sinusoidal behvior so that the same procedure could be repeated. As the techniques used were empirical, based on trial and error rather than mathematical rigor, no further details will be given. The final results are:

$$\begin{array}{l} f\left(x\right)=f_{0}\left(x\right)+a_{1}\left(x\right)\sin\left[\alpha_{1}\left(x\right)\right]\\ +a_{2}\left(x\right)\sin\left[\alpha_{2}\left(x\right)\right]+a_{3}\left(x\right)+\varepsilon \left(x\right), \end{array}$$ (5a)

$$\begin{array}{l} g\left(x\right)=g_{0}\left(x\right)+b_{1}\left(x\right)\sin\left[\beta_{1}\left(x\right)\right]\\ +b_{2}\left(x\right)\sin\left[\beta_{2}\left(x\right)\right]+b_{3}\left(x\right)+\varepsilon \left(x\right), \end{array}$$ (5b)

$$\left|\varepsilon \left(x\right)\right|<3\;10^{-6},\;0.6\leq \;x\leq 3.1,$$ (5c)

where

$$a_{1}\left(x\right)=\left(1.3384+0.2158x\right)10^{-3},$$ (5d)

$$a_{2}\left(x\right)=\left(6.158+0.073x\right)10^{-5},$$ (5e)

$$a_{3}\left(x\right)=\left(-15.604+2.88x\right)10^{-5},$$ (5f)

$$\alpha_{1}\left(x\right)=\left(3.0824+14.8294x+0.00462x^{2}\right)/\left(1+x\right),$$ (5g)

$$\alpha_{2}\left(x\right)=\left(0.857+11.78x+0.773x^{2}\right)/\sqrt[{3}]{1+2.354x^{2}},$$ (5h)

and

$$b_{1}\left(x\right)=\left(1.367-0.2278x\right)10^{-3},$$ (5i)

$$b_{2}\left(x\right)=\left(8.94-0.19x\right)10^{-5},$$ (5j)

$$b_{3}\left(x\right)=\left(-7.86+11.2x\right)10^{-5},$$ (5k)

$$\beta_{1}\left(x\right)=\left(16.442-0.00012x+0.00034x^{2}\right)\times \left(1-e^{-0.7738\sqrt{x}}\right),$$ (5l)

$$\beta_{2}\left(x\right)=\left(1.524+29.1x-0.39x^{2}\right)/\sqrt[{3}]{1+5.934x^{2}}.$$ (5m)

The digits appearing in these expressions are significant within the uncertainties stated in Eq. (5c). By substituting the numerical values of *f*(*x*) and *g*(*x*) given by these equations into Eqs. (1a) and (1b), and comparing the results to the exact values in Ref. [3], it was found that they yield *C*(*x*) to ± 3.5 × 10^−6^ for 0.6 ≤ *x* ≤ 3.1.

## 3. Application

The results of Sec. 2 were entered into a spreadsheet program and *C*(*x*) and *S*(*x*) were computed for 0.6 < × < 3 from Eqs. (1a) and (1b) and Eqs. (5a) to (5m). For the respective ranges *x ≤* 0.3, and *x* ≥ 3, the the Taylor series of Eqs. (3a) and (3b) and the asymptotic expansions of Eqs. (4a) and (4b) were used.
